# Divulging Patterns: An Analytical Review for Machine Learning Methodologies for Breast Cancer Detection

**DOI:** 10.7150/jca.118698

**Published:** 2025-10-20

**Authors:** Alveena Saleem, Muhammad Umair, Muhammad Tahir Naseem, Muhammad Zubair, Silvia Aparicio Obregon, Ruben Calderon Iglesias, Shoaib Hassan, Imran Ashraf

**Affiliations:** 1 Faculty of Information Technology and Computer Science, University of Central Punjab, Lahore, Pakistan.; 2 Department of Electronic Engineering, Yeungnam University, Gyeongsan, 38541, Republic of Korea.; 3 IRC-FDE, King Fahd University of Petroleum and Minerals, 31261, Dhahran, Saudi Arabia.; 4 Universidad Europea del Atlantico, Isabel Torres 21, Santander, 39011, Spain.; 5 Universidad Internacional Iberoamericana, Campeche 24560, Mexico.; 6 Universidad Internacional Iberoamericana, Arecibo, Puerto Rico 00613, USA.; 7 Universidade Internacional do Cuanza, Cuito, Bie, Angola.; 8 Universidad de La Romana, La Romana, Republica Dominicana.; 9 School of Computer Science and Engineering, Nanjing University of Science and Technology, Nanjing, 210094, Jiangsu, China.; 10 Department of Information and Communication Engineering, Yeungnam University, Gyeongsan, 38541, Republic of Korea.

**Keywords:** tumor detection, breast cancer, deep learning, segmentation

## Abstract

Breast cancer is a lethal carcinoma impacting a considerable number of women across the globe. While preventive measures are limited, early detection remains the most effective strategy. Accurate classification of breast tumors into benign and malignant categories is important which may help physicians in diagnosing the disease faster. This survey investigates the emerging inclination and approaches in the area of machine learning (ML) for the diagnosis of breast cancer, pointing out the classification techniques based on both segmentation and feature selection. Certain datasets such as the Wisconsin Diagnostic Breast Cancer Dataset (WDBC), Wisconsin Breast Cancer Dataset Original (WBCD), Wisconsin Prognostic Breast Cancer Dataset (WPBC), BreakHis, and others are being evaluated in this study for the demonstration of their influence on the performance of the diagnostic tools and the accuracy of the models such as Support vector machine, Convolutional Neural Networks (CNNs) and ensemble approaches. The main shortcomings or research gaps such as prejudice of datasets, scarcity of generalizability, and interpretation challenges are highlighted. This research emphasizes the importance of the hybrid methodologies, cross-dataset validation, and the engineering of explainable AI to narrow these gaps and enhance the overall clinical acceptance of ML-based detection tools.

## 1. Introduction

The cell is a basic structural and functional unit of an organism which consists of numerous cell organelles. During a biological clock that a cell undergoes, it continues to grow and experiences cell division (mitosis) after a specified period of time. A cell becomes malignant or cancerous when it loses its capability to stop cell division. Such unnecessary mitosis led to the cells accumulating at a particular location and time forming a mass known as tumor 1. Two kinds of tumors have been identified until now; benign means non-cancerous and malignant means cancerous. A cancerous tumor is malignant when it starts invading and damaging the nearby cells 2.

Breast cancer is a type of cancer that includes the cancerous tumor development in the tissues of the human breast. Every woman is at the peril of forming breast cancer at some stage of her life. The year 2020 observed the morbidity of more than four million women around the world 3 and the major reason for this wide-scale casualty is breast cancer. However, a significant number of them is gathered in third world countries which accommodate 72% cases. This death toll difference between economic- social areas has irked between 1990 and 2019 and this development is expected to proceed 4.

**Figure 1:**
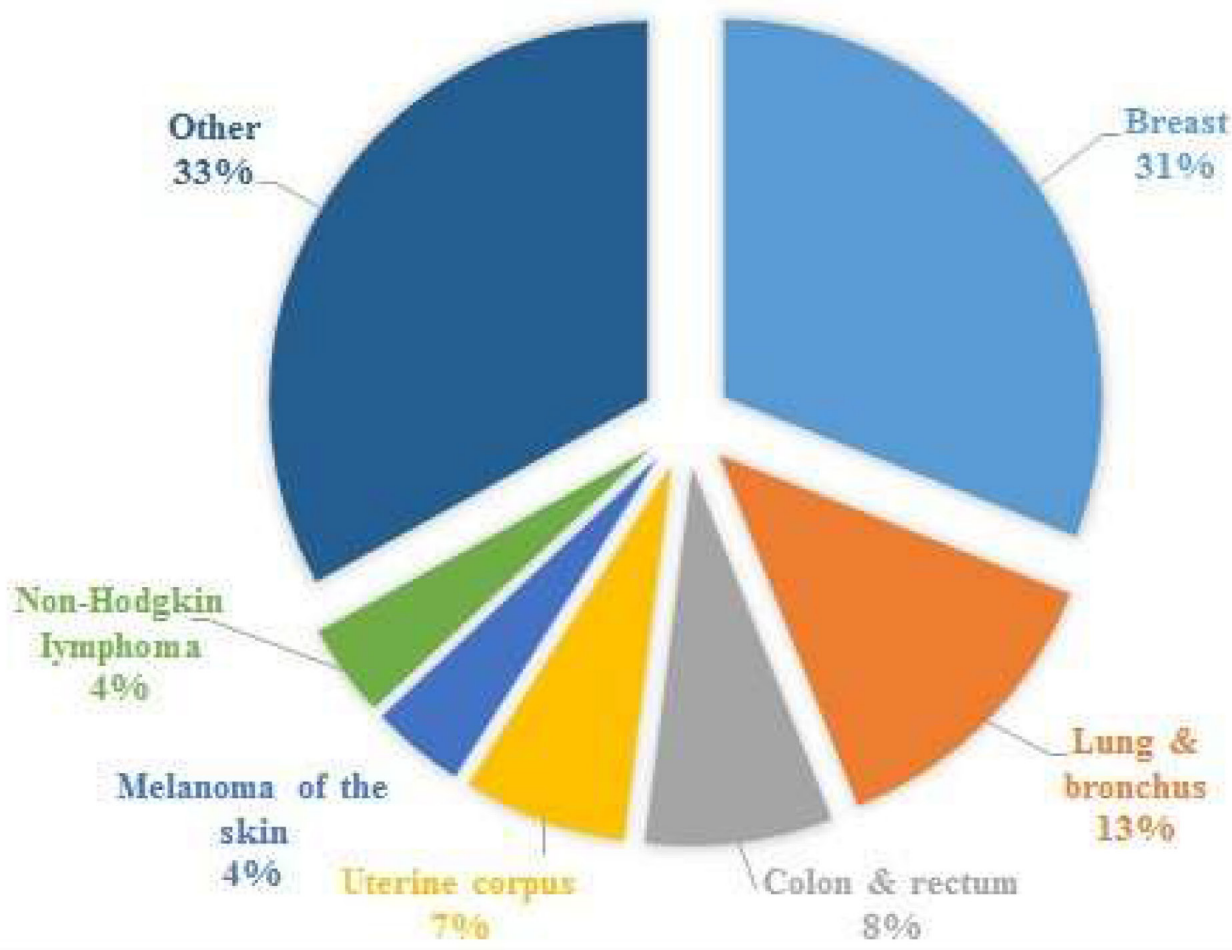
New cases in women for the year 2023, cancer statistics have been taken from 6.

According to Health at Hand 5, in 2020, globally breast cancer is the most dominating type of carcinoma, afterwards colon and rectum cancer. Figure 1 shows the number of new cancer cases in women for the year 2023, indicating the highest number of breast cancers, with other types such ovarian, lung cancer, colon cancer, etc. 6-8. These are the main cancer kinds across most countries; however, they vary with regard to their ranking across the world. According to The DAWN 9, Pakistan has the highest number of breast cancer cases in Asia, with an estimated 40,000 women falling victim to this fatal disease. Consultants at Shaukat Khanum Memorial Hospital connect the soaring incidence rate of breast cancer to Pakistan's orthodox societal norms and the lack of an advanced diagnostic system.

To mitigate the mortality rate of breast cancer, early detection is crucial and can be bolstered through accurate classification of breast cancer tumors into benign called non-cancerous or malignant called cancerous classes 10. Breast cancer has a considerable number of categorizations, which may help clinicians to recommend the best treatment. Binary classification or classification into two classes is most significant among them that is; whether the tumor is benign (non-cancerous) or malignant (invading the nearby cells) 11. At present, it is crucial to group the cancer tumor as the acuteness of the ailment is figured out by these sorts of classifications. Various studies have been carried out utilizing certain ML methodologies and different datasets for the purpose of classifying the cancer tumor as benign or malignant 12, 13. Such methodologies can help physicians to medicate the cancer properly. Over time, certain standard datasets have come to light in the literature that have been utilized by scientists for the diagnosis and prediction of breast cancer.

This review aims to address the gap in comprehending the latest trends and patterns in the evolution of breast cancer detection and the effectiveness of various detection methods, including deep learning (DL), feature selection-based, ensemble classifications, and image-based segmentation techniques. It further focuses on and evaluates the utilization and efficacy of a variety of datasets wielded while training breast cancer detection models, emphasizing their significance in improving detection accuracy.

### 1.1 Overview of Breast Cancer and its Societal Impact

In terms of women's deaths caused by cancer, breast cancer morbidity, and mortality numbers are considerable 3. In addition, the phase extends beyond the patient's physical health in talking about the emotional, social, and economic outcomes of the disease. Families and caregivers often have to deal with a great amount of stress, and the costs of treatment and long-term care spiral ever higher in our healthcare systems. Further, in low-resource areas, disparities in access to healthcare enhance outcomes as there is often late-stage presentation. One of the measures taken toward combating breast cancer was to improve medical imaging, make public breast cancer awareness, and set up screening programs. These steps are being taken, but diagnostic inaccuracies persist, with high rates of false positives, and a need for expert interpretation. ML can address these limitations and represents real transformative potential to deliver precise, automated, and scalable detection and diagnosis solutions 4.

### 1.2 Challenges in Diagnosis and Treatment

The diagnosis of breast cancer and treatment includes several challenges that influence the accuracy, efficacy, and accessibility of watchfulness. Detection techniques mostly encounter certain limitations because of technological obstructions, human inference problems, and heavy costs 14, 15. Likewise, therapeutic approaches should account for tumor diversity, individualized responses, and prospect consequences, creating personalized treatment intricate. Tackling such challenges necessitates a blend of enhanced diagnostic techniques, advanced care methods, and the integration of innovative and modern technologies like ML 16.

#### 1.2.1 Limitations of Conventional Diagnostic Approaches

- **Heavy Cost and Restrained Approach:** Modern imaging approaches like MRI and 3D techniques are costly and may not be accessible in resource-constrained environments ultimately leading to inequalities in early diagnosis 4, 16.
- **False Negative and False Positive Cases:** The mammograms may create false-positive outcomes that can lead to unwanted biopsies, whereas false negative results linger the treatment, which minimizes the chances of early detection and care 17.
- **Bias in Interpretability:** Oncologists' evaluation can oscillate on the basis of experience and training resulting in unpredictability in the diagnosis 18.
- **Radiation Subjection:** The ionizing radiations can be subjected to the patients with frequent mammograms resulting in the anticipated risks 17.
- **Trouble in the Detection of Thick Breast Tissue:** Thick breast tissue can cloak the tumors in mammograms, making it even more challenging to detect malevolence on time 5, 16.

### 1.3 Research Questions

For this review, we formulated the following research questions.

- How is the performance of various ML models impacted or inflicted by the dataset choice such as WDBC, BreakHis, etc. in the diagnosis of breast cancer?
- Do the research results contain the prejudice just because of the excessive utilization of prevailing datasets and how this can be alleviated?
- What are the determinants that impact the selection of algorithms for the fact finder in regard to breast cancer detection?
- Is there any trade-off between interpretation and accuracy while selecting the algorithm for diagnosis of breast cancer?
- What will be the future trend of this research? i.e. How can the simpler algorithms such as Logistic regression hl(LR) in contrast to the more complex models like DL with respect to computational cost and accuracy?

### 1.4 Research Objectives

- To analyze the application of ML techniques in breast cancer detection, we look into the role segmentation-based as well as feature selection-based methods play in enhancing diagnostic accuracy and efficiency.
- To identify widely used datasets and popular ML methods, public and benchmark datasets widely used in breast cancer research and ML techniques (supervised learning, ML, ensemble methods) will be surveyed.
- To evaluate performance metrics used for comparison, the survey attempts to standardize the notion of comparing the efficacy of various ML models, by evaluating the indicators such as accuracy, precision, recall, F1-score, and area under the curve (AUC).
- To highlight limitations and propose future directions for research, these issues include data scarcity, model interpretability, and computational requirements, and we provide suggestions to help serve to advance the field.

### 1.5 Rationale of this Survey

The extending intersection among ML and breast cancer detection provides the trans- formative potential in early detection, the most efficient strategy for mitigating rates of mortality. ML techniques can significantly improve the diagnostic accuracy, speed, and reliability.

In spite of the growing volume of ML-based research, various existing researches are fragmented focusing narrowly on specific algorithms, datasets, or imaging modalities.

This shattered landscape poses various challenges for the researchers and clinicians in recognizing the effective strategies, comparing results, and building upon prior work. This survey tackles these challenges by aiming to:

- Produce the recent developments in ML-based breast cancer diagnosis.
- Evaluate the strengths and shortcomings of various methodologies.
- Highlight frequently utilized datasets and their impact over model performance.
- Explore the role of Explainable AI (XAI) in enhancing clinical trust.
- Provide structured insights for researchers, developers, and clinicians seeking to develop transparent and efficient diagnostic solutions.

By consolidating existing findings into a unified narrative, this survey helps improve reproducibility, inform practical decision-making, and identify promising areas for future research.

### 1.6 Why This Research is Significant

Breast cancer persists as a major reason for cancer-related casualties among women throughout the world. The efficacy of treatment is highly contingent on early and precise diagnosis, while conventional diagnostic approaches often suffer from certain constraints, including high false positives and false-negative rates, bias in interpretability, and availability issues. ML has come up as an auspicious tool in clinical diagnostics, offering top accuracies, scalability, and automation. However, in spite of substantial advancements, ML-based breast cancer diagnostics still encounter challenges in terms of quality of data, generalizability of a model, and medical adoption. This sur- vey attempts to provide a comprehensive overview of various ML approaches utilized in breast cancer detection, pointing out their strengths, constraints, and potential improvement areas. Table 1 presents comparative analysis of existing surveys.

**Figure 2:**
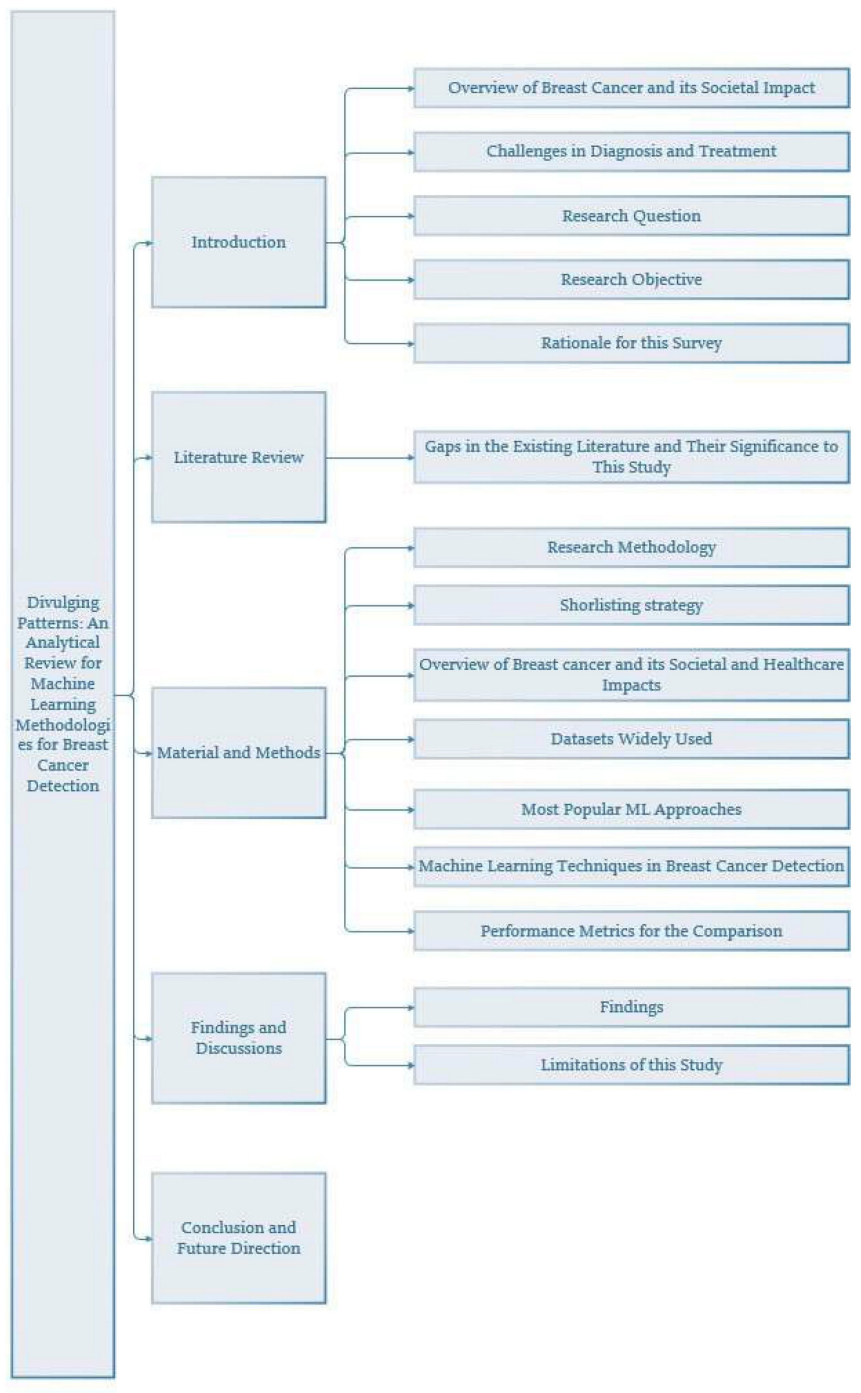
Structure of the paper with section and subsections.

Figure 2 shows the organization of the sections that follow the introduction. After the literature review in Section 2, the methodology is presented in Section 3. Findings and discussions are given in Section 4 while the conclusion and future research directions are presented in Section 5.

## 2. Literature Review on Breast Cancer Detection

A considerable number of ML methodologies have been employed so far to correctly diagnose breast cancer disease in various research. In 25, a Tabu search was done to choose the most appropriate features from the dataset for the detection of breast lesions or tumors using a rough set. The method was tested on the WDBC and BIDMC-MGH datasets. AdaBoost, hlK-Nearest Neighbor (KNN), and hlLR were used as performing models. hlKNN achieved the highest accuracy among all using Tabu search at 98.24%.

hlLR, KNN, discrete cosine transform (DCT), random forest (RF) classifier, hlSVM, multilayer perceptron (MLP), and ensemble MLP with genetic algorithm (GA) have been applied in 26 using the WBCD dataset. The study accomplished an accuracy of 98% with MLP-GA and holdout approach while 99.7% using MLP- GA and cross-validation. In 27, an artificial neural network (ANN) was optimized by integrated artificial immune system and artificial bee colony (IAIS-ABC-CDS), momentum-based gradient descent backpropagation (MBGD), simulated annealing (SA), resilient backpropagation techniques (RBPT) and GA approach on to the publicly available WBCD dataset for breast cancer detection. The study achieved an accu- racy of 99.34% using IAIS-ABC-CDS with MBGD and 99.11% using IAIS-ABC-CDS with RBPT.

Bayesian classifier-embedded integrated genetic-driven framework, GA, kernel- based Bayesian classification was applied by Wuniri et al. 48 on the WDBC dataset for the diagnosis of breast cancer attaining 97.1% accuracy. Abunasser at el. 28 utilized DL model Xception over BreakHis dataset collected from the Kaggle repository and achieved the accuracy of 99.78% for training, 98.59% for validation, and 97.60% for testing. Additionally, the Xception model showed a precision of 97.60%, recall of 97.60%, and an F1 score of 97.58%. In 29, authors applied hlCNN to the BreakHis dataset for the accurate diagnosis of breast cancer and secured the training accuracy of approximately 99% and testing accuracy of 97.80%.

The study 30 demonstrates the application of a hybrid gravitational search optimization algorithm and emperor penguin optimization (HGSAEPO) for the feature selection while RF, SVM, LR, decision tree (DT), and KNN for the classification of breast tumor into benign or malignant categories. The accuracy was 98.31% with HGSAEPO and RF showing a 97% sensitivity, 98.87% specificity, 98% precision, and 95.39% F1 score. Kadhim et. al 31 performed the comparison of different ML techniques comprising of DT, quadratic discriminant analysis, AdaBoost, bagging meta estimator, extra randomized trees (ERT), Gaussian process classifier, Ridge, Gaussian Naive Bayes (GNB), KNN, MLP, and hlSVM classifier. The authors found out that on the WDBC dataset, a 97.36% accuracy was achieved in the case of ERT which outperformed other algorithms for breast cancer diagnosis.

The authors employed ANN in 32 for breast cancer detection through WBC and WDBC datasets securing an accuracy of 99.85% on WBCD and 99.47% on WDBC. In 33, Yusuf et. al described LR, SVM, RF, gradient boost (GB), and AdaBoost hl(AB) for the classification of breast cancer tumors into benign and malignant categories using the WDBC dataset achieving the accuracy of about 99% with LR, RF, and hlAB. Rakibul et al. 34 employed LR and SVM including linear SVM (LSVM), and quadratic SVM (QSVM) to WBCO, WDBC, and WPBC datasets and attained the accuracy of 94% for WBCO, 97.4% using QSVM on the WDBC dataset, and 83.5% using LR on the WPBC dataset.

In 35, wrapper subset selection method, correlation analysis, and principal component analysis (PCA) are used for feature selection and NB, SVM, DT, KNN, RF, LR, stochastic gradient descent learning-based ensemble classification methodology for breast cancer diagnosis is adopted. A 98.24% accuracy was achieved using the WDBC dataset. Huang Z and Chen D. A 36 applied variable importance measure (VIM), hierarchical clustering RF algorithm, DT, hlAB, and RF models on WBCD and WDBC datasets with accuracy of 97.05% on WDBC, and 97.76% on WBC with HCRF. KNN, chi-square-based feature selection, L1 based selection from model feature selection are applied in 37 on the WBCD and WDBC datasets having an accuracy of 99.42% for WBC, and 99% for WDBC dataset with L1-based feature selection.

Dragonfly algorithm (DA), PCA, DL models, SVM, RF, and KNN were utilized by Ibrahim et. al. 38 for breast cancer detection and achieved 97.90% accuracy. In 39, Akkur et al. used relief and binary Harris hawk optimization (BHHO) hybrid model, KNN, SVM, LR, and NB for the diagnosis of breast cancer using the WDBC, WBCD and mammographic breast cancer dataset (MBCD). They secured an accuracy of 98.77% for the WDBC dataset. For the WBCD, 99.28% accuracy and for MBCD 97.44% accuracy was secured with relief-BHO-SVM. Ensemble filter-based feature selection with 1-D CNN (1D-CNN) was employed in 40 with an accuracy of 98.5% via the WDBC dataset.

In 41, the WDBC dataset was utilized for breast cancer detection through Pear- son's correlation coefficient, lasso, and minimum redundancy-maximum relevance (mRMR) for feature selection and SVM, light GBM, RF, DT, NB, KNN, LR were used for the classification of breast tumor into benign and malignant classes. Hossin et. al in 42 performed a comparison of different ML algorithms using univariate feature selection, recursive feature elimination, correlation heatmap, LR, RF, KNN, DT, hlAB, SVM, GB, and Gaussian NB. They found that LR and SVM are more effective as they attain an accuracy of 99.12% on the WDBC dataset. In 43, Sundar and the co-authors utilized the ResNet50v2 model of CNN and ensemble approach with DT, RF, ET, and XGBoost on invasive ductal carcinoma (IDC). The ensemble model achieved an accuracy of 99.82%.

The study 44 is associated with the usage of SVM and its parameters' fine-tuning for the diagnosis of breast cancer using WDBC attaining an accuracy of 95.61%. Doaa et al. in 45 utilized thermal images from the DMR-IR dataset and employed Gabor filters, canny edge detection, and holistically nested edge detection, CNN, RESNET- 50, SVM, and XGB achieving 96.23% accuracy. Saurav and co-authors utilized TCGA and applied RF, SVM, DT, KNN, Gaussian NB, and XGBoost in 46 and got 97.19% accuracy. While in 47, XGBoost was used on the WDBC dataset and the resulting accuracy was 99.12%.

### 2.1 Gaps in Existing Literature and Their Significance to This Study

In spite of significant breakthroughs in the ML area for breast cancer detection, various gaps in the existing literature hamper its full prospective in medical implementations. These gaps comprise:

- **Finite Generalizability and Dataset Diversity:** Mostly ML models to detect breast cancer depend on some publicly accessible datasets, such as WDBC, WBCD, and BreakHis 25-33, 48, restricting the generalizability of ML models. Such datasets are usually less diversified in regards to the demographics of patients, imaging modalities, and the subtypes of tumors, which can cause prejudiced models that struggle to perform on real-time clinical data.
- **Contribution of this Study:** This survey highlights the significance of cross- dataset validation and hybrid or ensemble learning methodologies to enhance generalizability and make sure that ML models are robust in nature across various populations and clinical settings.
- **Over-Dependency over Black-Box Models:** DL approaches, specifically CNNs have exhibited top accuracy in breast cancer detection 28, 29, 34, 35. However, they often lag behind in terms of interpretability and explanation due to which they are not trustworthy and reliable making them difficult to get accepted and adopted in clinical settings.
- **Contribution of this Study:** This survey highlights the importance of XAI techniques to improve transparency, and to make sure that ML-driven methodologies are interpretable and clinically adaptable.
- **Lack of Balance Among Accuracy and Interpretability:** Most of the studies prefer accuracy over model interpretability 36-38 making it challenging for clinicians to trust and rely on the predictions of the model. Conventional models such as hlLR and hlDTs provide good interpretability but are not highly accurate.
- **Contribution of this Study:** This research explores certain hybrid methodologies through the analysis of both segmentation-based and feature selection-based approaches. This equalizes accuracy and interpretability to make reliable detection methods.
- **Scarce Systematic Performance Metrics:** Existing literature often utilizes various performance metrics such as accuracy, F1 score, AUC-ROC, etc. 39-41 that makes it challenging to contrast models equitably. In addition to this, some research reports high accuracies through training data with no meticulous validation on test datasets.
- **Contribution of this Study:** This survey supports the systematic evaluation standards and benchmarking approaches to ensure unbiased and staunch contrast between various ML models.
- **Absence of Comparative Analysis:** Many research works 42, 43, 49 analyze specific ML approaches without providing a comprehensive contrast of segmentation-based and feature selection-based classification methodologies.
- **Contribution of this Study:** This research offers a structured juxtaposition of certain ML models, even containing DL and ensemble approaches.
- **Difficulties in Medical Integration and Acceptance:** In spite of the success of breast cancer diagnostic based on ML in terms of research, they face hindrances in adoption by clinical professionals because of administrative challenges, data privacy issues, and the requirement for comprehensive validation as well as controlling, ethical, and computational limitations 50-52.
- **Contribution of this Study:** This research explores prospective resolutions, like ensemble learning and administrative-compliant ML models, to narrow the gap between research development and real-time clinical applications.

By tackling these gaps, this study aims to offer practical comprehension and facts for researchers and professionals in the domain. It advocates for the development of reliable, robust, interpretable, and clinically acceptable ML models for the detection of breast cancer, facilitating enhanced detection accuracy, early diagnosis, and better medical outcomes.

## 3. Material and Methods

### 3.1 Research Methodology

**Figure 3:**
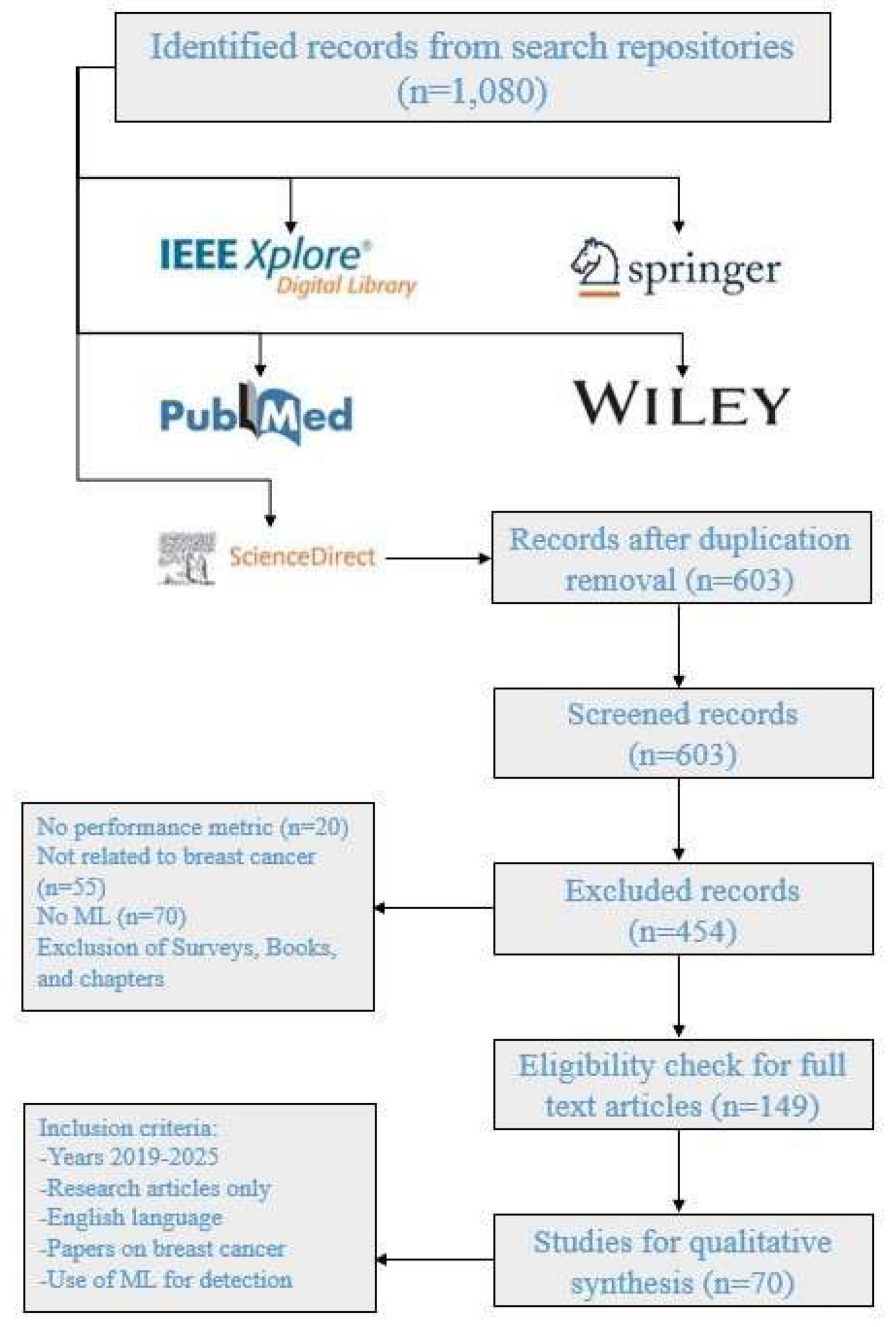
PRISMA approach for this review.

This research offers streamlined approaches for scrutinizing, classifying, and amalgamating the literature commensurate with the established objectives. This emphasizes the spheres that may set out as a strategy for anticipated research inclination in the particular domain. This survey has been carried out in a number of steps. The first step comprises the research question definitions, while in the second step, the research objectives have been developed using the pre-defined research questions. In the third step, the shortlisting strategy is formulated to find out the related articles after which they will get nominated, categorized, and scrutinized in conjunction with the research domain. Finally, the results were discussed and analyzed as per research questions. Figure 3 presents the adopted approach for this review.

### Shortlisting Strategy

The articulation of a search proposition to gather the related and original information within the specified area is the most critical step in the formulation of this review. This research examines relevant literature from 2019 to 2024, collected from a number of databases such as MDPI, IEEE, Elsevier, Springer, Neural Network World, and Computational and Mathematical Methods in Medicine. The relevant journals have higher H-index and good citation rates and consulted with specific keywords such as “machine learning,” “breast cancer detection,” “segmentation-based classification,” and “feature selection”, These keywords were used to identify relevant studies in academic databases like PubMed, Wiley, Springer, etc.

**Figure 4:**
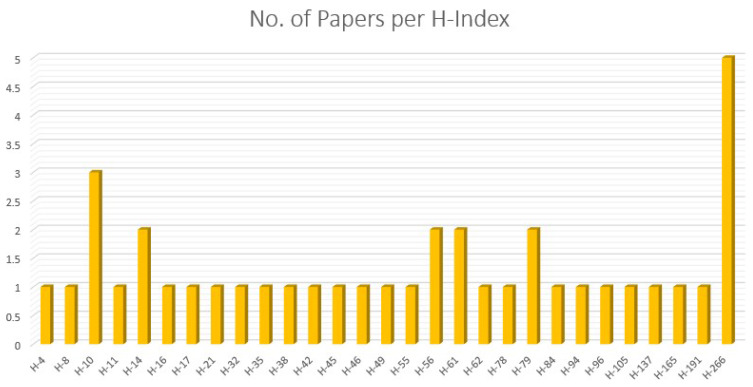
Number of papers per H-index.

Applying the search string to the diverse digital repositories resulted in the acquirement of a huge amount of data, which needed to be shortlisted by going through a multi-stage shortlisting process. The research papers were selected on the basis of an H-Index criterion, Figure 4, and restricted to the publications from 2019 till 2024, Figure 5. After the removal of redundancy, the papers were scrutinized via abstract reading as well as results evaluation so that the most relevant articles were selected.

Benchmark datasets that are readily available to the public were used in shortlisted studies (e.g., WBCD, DDSM), such that results are reproducible. The experimental results focus was the ML techniques discussions, and the performance metrics reported were used to evaluate the articles. Studies with more complete experimental validation and comparison were preferred. Research papers across a wide range of ML techniques from traditional supervised learning to DL were included as part of the reporting effort to maintain a balanced review. Through adopting this systematic shortlisting approach, the survey conducts comprehensive and impartial scrutiny of the state-of- the-art ML methodologies applied for breast cancer detection.

#### 3.2.1 Inclusion Criteria

The following kinds of research and datasets were incorporated in this survey.

- **Dataset Connectedness:** Researches or sources that particularly used breast cancer datasets, including Histopathological images, Mammograms, Thermal images, Clinical data (e.g., reports, tabular data)
- **Public Available:** The studies utilizing datasets which are publicly accessible were included.
- **Real time Individual Data:** Real patient data incorporated in the literature was given preference.
- **Language:** Articles published in English were chosen.
- **Time frame:** Research articles published from 2019 to 2025 were selected.
- **Use of ML:** Priority was given to the studies using ML or DL approaches in conjunction with breast cancer detection, diagnosis, or prognosis.

#### 3.3.2 Exclusion Criteria

The following kinds of research were excluded while shortlisting.

- **Non-Dataset Articles:** Publications that included the discussion of breast cancer but did not incorporate any datasets, were excluded.
- **Restricted Access Data:** Studies including datasets not publicly accessible for academic or research use were excluded.
- **Redundant Research:** Duplicate entries or multiple studies on the same topic were filtered out to avoid repetition.
- **Surveys, Books, Magazines:** Surveys were only included for the purpose of comparison, however, books and magazines were completely omitted.
- **Not Related to ML or Breast Cancer:** Studies of pure medical nature meaning that it does not incorporate ML for breast cancer detection were excluded. As well as, the research including cancer other than breast cancer or any other disease was also omitted.
- **Language Hurdle:** Researches published in languages besides English were excluded.

Table 2 shows the distribution of selected papers with respect to the publisher.

### 3.3 Breast Cancer Overview

Breast cancer is one of the prevailing cancers across the world, influencing a wide range of individuals perennially. It is attributed to the unhampered or abnormal division of malicious cells in breast tissues, together they become a malignant or cancerous tumor invading the surrounding cells. Although this cancer primarily targets women, men can also become a victim of it yet at minimum frequency. As per the World Health Organization (WHO), the predominant cause of cancer-related casualties in women is breast cancer, with substantial distinctness in the rate of occurrence and death toll worldwide because of inequalities in healthcare availability, cognizance, and early diagnosis programs.

#### 3.3.1 Societal and Healthcare Impacts of Breast Cancer

Substantial psychological distress including anxiety, fear of recrudescence, financial stress, and depression for the patient and their families is caused when breast cancer is diagnosed. The diagnosis of breast cancer often leads to a financial burden for low- income families as the cost associated with the surgeries, radiation, chemo-therapies, and related treatment is significant. The screening programs are scarce in third-world countries leading to the diagnosis at the last stage of cancer. Many people in such countries owing to the disparities are reluctant to regular scan of breast tissues due to the cost associated with it leading to distress and suffering.

A multidisciplinary approach is required for the treatment of breast cancer involving a versatile number of surgeons and oncologists which impose a substantial burden on healthcare systems. However, countries having early detection tools and well-established screening systems have higher chances of survival as compared to third-world countries where these facilities are scarce and the available programs are costly for log-wage families. So, there is a need for early detection including scalable and cost-effective AI-driven solutions for the diagnosis of breast cancer.

#### 3.3.2 Early Detection is Imperative

Lowering breast cancer mortality rate and improving the health of breast cancer patients depend on early detection. However, if diagnosed early enough, there is a 90 out of 100 chance the person will survive breast cancer. These limitations do not preclude the use of ultrasound or mammography, classic diagnostic tools that suffer however operator dependency and the concomitant variability of its interpretation, failing to satisfy needs in underserved areas. What this means is that early detection and the capability of doing so are being greatly enhanced by ML as a powerful tool. Large datasets are used by ML models to uncover patterns and anomalies that a human observer might miss. This is a characterization of malignant tumors at a greater speed and with accuracy, facilitated by the use of image segmentation, feature extraction, and predictive modeling techniques. On top of these, ML-based tools can further filter out high-risk prospective patients, grade a case's urgency, and help clinicians update their diagnostic workflow and corresponding patient outcomes.

## 4. Findings

This section describes the conclusions and key findings attained after analyzing the 40 publications selected in this survey. All RQs are briefly described in order to clarify the respective exploring areas of the breast cancer detection domain.

### 4.1 Datasets Widely Used

Different publicly available datasets have largely contributed to the advancement of ML and DL techniques for breast cancer detection. These datasets differ in size, imaging modalities, annotations, and patient demographics, enabling researchers to develop and evaluate diverse models.

**Figure 6:**
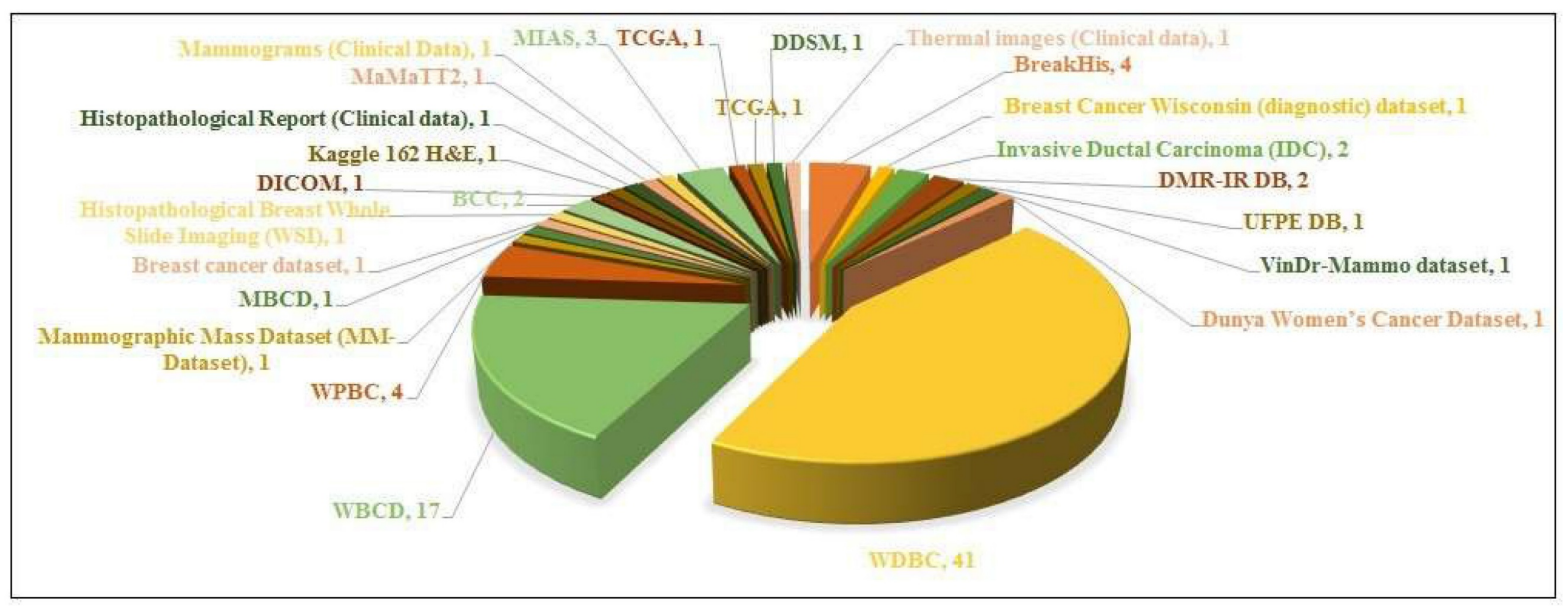
Number of research papers per dataset.

The datasets that have been widely utilized in the study are WDBC and WBC datasets as illustrated in Figure 6. This chart demonstrates the popularity of certain datasets in breast cancer detection literature.

- The WDBC dataset is the commonly implied dataset, with 26 papers referencing it. This points out its importance in the research and its priority as a standard dataset.
- The WBC is the second most widely cited dataset, to be referenced in 14 papers, proving its significance for the study of breast cancer diagnosis.
- Other datasets i.e. the mammographic mass dataset (MM-Dataset) and Histopathological breast whole slide imaging (WSI), are of the least usage, referenced in about 3 or minimal studies.
- Other datasets, such as VinDr-Mammo Dataset, DMR-IR DB, UPFE DB, etc. have been referenced in only 1 paper each, illustrating their restricted incorporation in the research.

Conclusively, the supremacy of WDBC and WBC emphasizes their powerful dominance in the literature of breast cancer detection, probably because of their fine data, availability, and continuous domination. Other datasets which have been least employed so far are either new or less accessible to the general public which contributes to their limited application.

**Figure 7:**
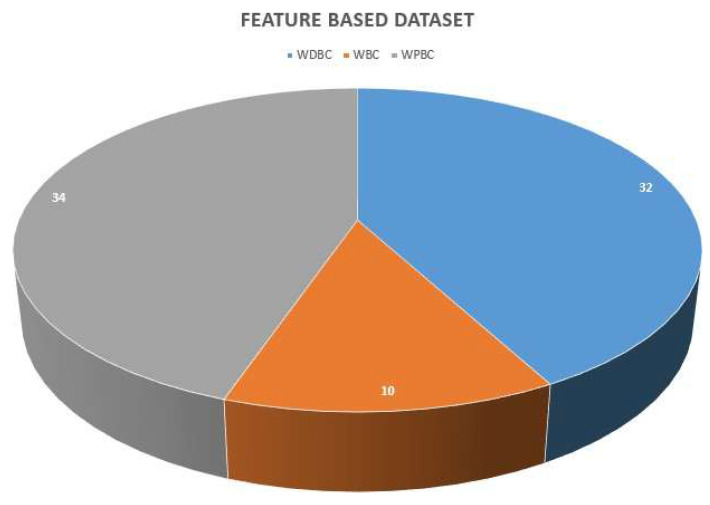
Size of feature-based datasets.

The pie chart in Figure 7 represents the apportionment of the datasets utilized for breast cancer diagnosis using various ML methodologies, partitioned into three classes: WDBC, WBC, and WPBC. Following is a comprehensive analysis of the figure targeting its relativity to breast cancer detection via ML approaches.

- **WDBC dataset:** This dataset is represented by the blue color section, and it comprises 32 instances, which makes it at the leading edge for the publicly available data.
- **Purpose:** The WDBC dataset is usually applied in ML methodologies for the classification of breast tumors into benign or malignant categories, helping to meticulously detect breast cancer.
- **WBC dataset:** This is the smallest dataset, illustrated in orange, and encompasses merely 10 samples.
- **Purpose:** This dataset presents an underlying principle for the fundamental level ML algorithms in breast cancer diagnosis, mainly utilized in the investigation calling for relatively smaller datasets.
- **WPBC dataset:** Shown in gray shade, this is the largest dataset containing 34 samples.
- **Purpose:** WPBC is indispensable for prognostic modeling, assisting the ML models in predicting cancer recrudescence and patient results in the longer run.

#### 4.1.1 Significance of Datasets

These datasets are widely utilized for the purpose of classification, prediction, and feature extraction or selection. WDBC and WDBC due to their enormous sizes are far suited for the diagnosis and prognosis scenarios.

A huge number of samples of these datasets contribute to the extrapolability of the ML models trained on these datasets. However, WBC due to its smaller size is not well suited for training complex or large-scale ML models but can be efficiently utilized for fundamental-level ML models.

**Figure 8:**
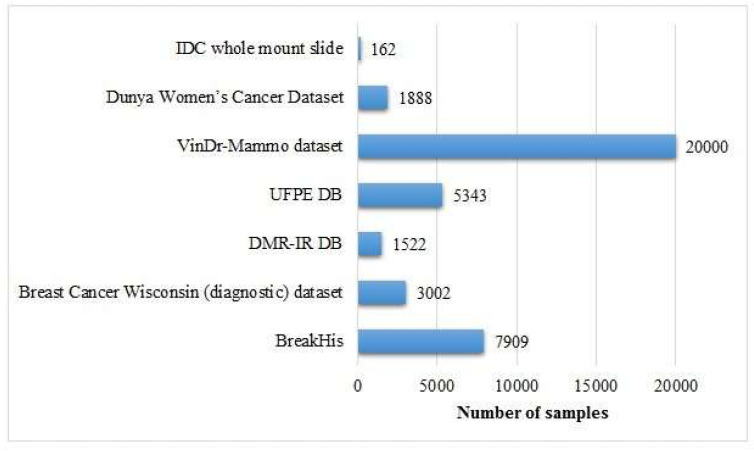
Size of image-based datasets.

The given chart in Figure 8 demonstrates the sizes of certain datasets based on images applied in breast cancer detection literature, presenting an analysis of their spectrum and scope. Following is a detailed overview:

#### i. BreakHis

- **Size:** This is a huge dataset utilized in this contrast, comprising approximately 60,000 images.
- **Importance:** Due to its substantial magnitude, BreakHis is extremely appropriate for the application of DL, which enables the training and performance evaluation of a robust ML model. The dominance of this dataset in the study reflects its large-scale usage and trustworthiness.

#### ii. VinDr-Mammo Dataset

- **Size:** This dataset comprises nearly 20,000 images.
- **Importance:** This dataset is comparatively sizable, which makes it invaluable for the development and testing purposes of advanced image-based breast cancer detection tools, specifically for mammography-based breast cancer studies.

#### iii. Breast Cancer Wisconsin (Diagnostic) Dataset

- **Size:** Comparatively smaller, with only a few thousand images.
- **Significance:** Although this is not as large as the BreakHis dataset, it is largely utilized because of its well-designated and premium attributes, which makes it a crucial resource for diagnosis of breast cancer detection.

#### iv. Dunya Women's Cancer Dataset

- **Size:** This is comparatively smaller, consisting of fewer thousands of images.
- **Importance:** Its small size makes it suitable for specific research as compared to it may be used for more specific or focused studies rather than high-level modeling purposes.

#### v. Invasive Ductal Carcinoma (IDC)

- **Size:** This dataset encompasses a few number of images.
- **Importance:** In spite of its relatively small size, It is invaluable for the focused research on invasive ductal carcinoma, a prominent sort of breast cancer.

#### vi. UPFE DB and DMR-IR DB

- **Size:** Both of these datasets are very small in size, probably less than 1,000 images.
- **Importance:** These datasets are likely utilized for small-scale or preliminary research because of their smaller size.

#### 4.1.2 Key Observations

BreakHis and VinDr-Mammo are two huge datasets, which makes them ideal for data- driven approaches such as DL. Small-scale datasets, like the Dunya Women's Cancer Dataset, IDC, UPFE DB, and DMR-IR DB, are suitable for focused research purposes or pilot studies. The size of the dataset performs a significant role in determining its applications, with large-scale datasets advocating the advanced techniques while smaller ones facilitating the targeted research. This apportionment highlights the significance of the selection of datasets on the basis of the study objective, with larger datasets like BreakHis being essential for high-level research and smaller ones serving as the focused ones. Figure 8 shows the size of image-based datasets.

### 4.2 Most Popular ML Approaches

**Figure 9:**
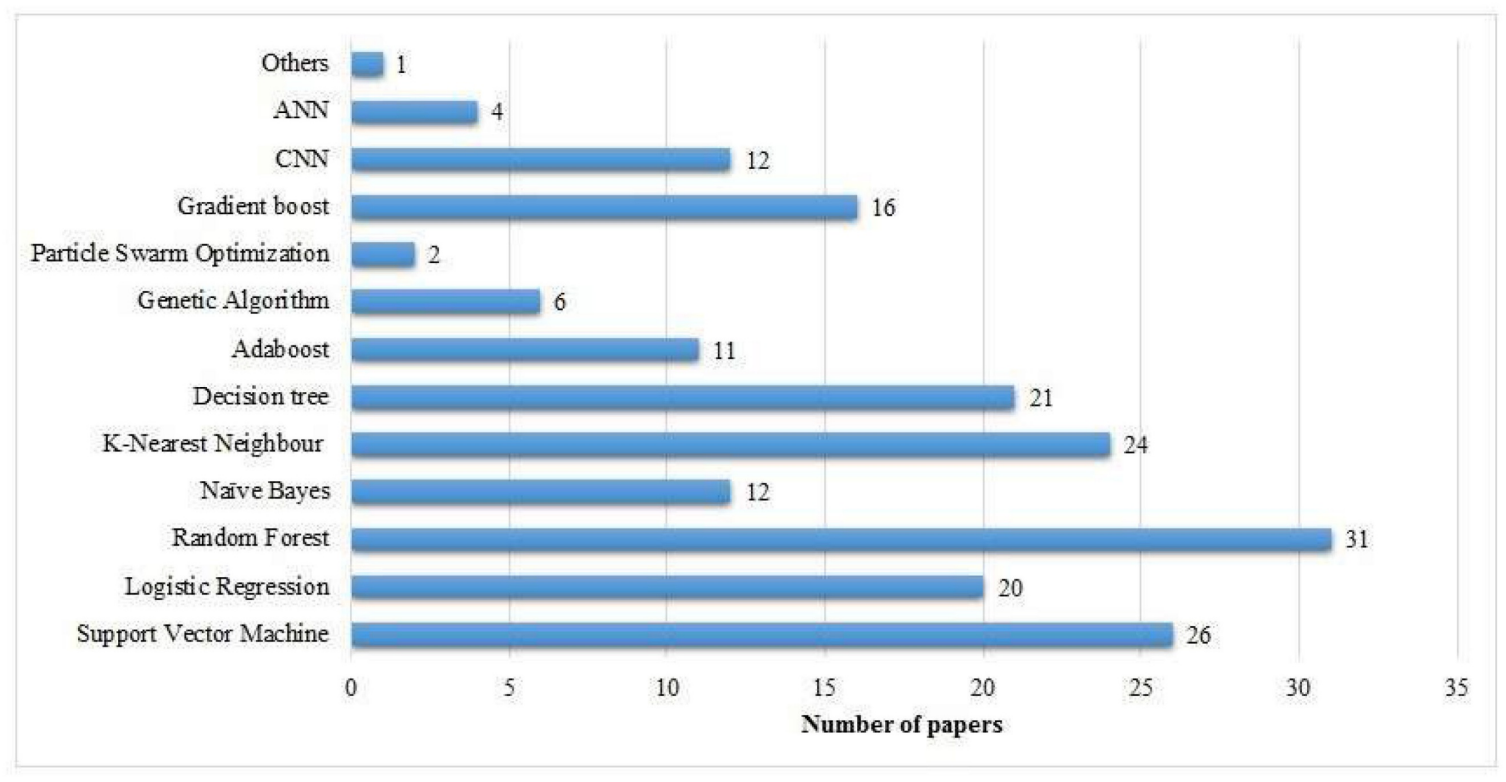
Most popular ML approaches in the literature.

Approaches such as SVM, RF, DTs, and KNN have a huge number of applications for the binary classification of breast cancer tumors as is shown in Figure 9.

CNNs have been shown to be state-of-the-art in medical imaging data analysis and work in the best interest of image data classification.

- **RF algorithm (20 papers):** This classifier has become the most cited one in breast cancer detection research. It is of great importance due to its ensemble learning abilities, which offer vigorousness and high accuracy for the classification of complex datasets.
- **KNN (16 papers):** KNN is the second-most widely utilized algorithm, probably due to its lucidness and efficacy for small-to-medium-level datasets.
- **LR (15 papers):** This conventional statistical classifier retains its significance, specifically in less complex or smaller datasets where lucidity is critical.
- **SVM (14 papers):** SVM is commonly given priority for its capability to perform well in binary classification methods as well as to handle high-dimensional data effectively.

#### 4.2.1 Moderately Popular Methods

Besides the widely used models like RF, KNN, etc, some ML models are moderately used in existing literature.

- **NB (12 papers):** The probabilistic and less complex nature of this dataset makes it efficient for initial research in breast cancer diagnosis.
- **GB (16 papers):** This model constructs complex and powerful classifiers by the combination of inconsistent or basic learning models. It is widely being embraced because of its robustness in manipulating and managing unbalanced or high-dimensional datasets.

#### 4.2.2 Impending or Less Commonly Used Approaches

A few ML models have been used in a few studies for breast cancer detection.

- **DL models (3 papers), hlCNN (4 papers), and ANN (4 papers):** Although these are least commonly referenced, however, these approaches are popularly bulging for breast cancer detection using image-based datasets, specifically along with the development in medical imaging methodologies.
- **GA (7 papers):** Traditionally applied for the purpose of feature selection or dimensionality reduction, this approach assists in improving the performance and efficacy of the classification models.
- **Particle Swarm Optimization (PSO) (2 papers):** It is a metaheuristic methodology mostly applied for the optimization of the model parameters feature selection.
- **AB (11 papers):** Adaboost is an ensemble approach widely applied for enhancing the performance of fundamental or simpler classifiers.
- **Recursive feature elimination (3 papers):** Mainly employed for feature reduction or elimination tasks, it assists in refining or moderating the features for classifier models.

#### 4.2.3 Applications of the Methods

- **Image Processing and Preprocessing:** Techniques such as CLAHE, YOLOv5, and Autoencoders are employed to enhance image lucidity or detection of breast tumors from mammographic images.
- **Feature Selection and Dimensionality Reduction:** Approaches such as GA, PCA, and Relief algorithm are utilized for refining the input features to enhance the performance of the model.
- **Classification and Diagnosis:** Methodologies like SVM, RF, KNN, and LR are employed for the classification of breast cancer as benign or malignant.
- **Optimization:** Algorithms such as PSO and GA are utilized for improving or tuning the parameters of the model or classifier to enhance the prediction and detection accuracy of the models.

### 4.3 Machine Learning Techniques in Breast Cancer Detection

Using ML to automate diagnostic processes in breast cancer detection has advanced its detection vastly. They are able to process massive medical imaging and patient data to find hints at malignancies. ML techniques broadly fall into two categories: The methods that I explore are segmentation-based classification and feature selection- based classification. Analysis of medical datasets using these two approaches can lead to unique advantages in the understanding of cancer, and their integration into clinical workflows will bring transformative improvements in cancer care.

#### 4.3.1 Segmentation-based Classification

Isolating regions of interest (e.g., tumors) from medical images as preprocessing is an important task, termed segmentation. This focuses on relevant anatomical structures and therefore enhances the reliability of subsequent diagnostic processes in case of accurate segmentation.

#### 4.3.2 Key Techniques and Applications

- **hlCNNs:** Spatial hierarchies can be captured by CNNs, making them the gold standard method of choice for image segmentation tasks. We demonstrate unusual success in segmenting mammograms and MRI scans using architectures such as U-Net and Mask R-CNN.
- **Fully Convolutional Networks (FCNs):** Specifically, FCNs are built for pixel-wise classification tasks and are particularly suitable for medical image segmentation. The images can have different resolutions and they can scale and adapt to different datasets.
- **Semi-supervised and Unsupervised Methods:** Semi-supervised techniques (i.e. GANs (Generative Adversarial Networks) and unsupervised clustering) can be used for segmentation of regions of interest in cases where the labeled data is scarce.

Radwan et al. 17 used YOLOv5, MedSAM segmentation models and contrast- limited adaptive histogram equalization (CLAHE) algorithm along with a Gaussian blur, ensemble deep random vector, functional link neural network algorithm for breast cancer diagnosis. While Sarfaraz et al. 16 applied H and E staining, Nuclei segmentation, nuclei-based instance segmentation as well as PCA and PSO for feature selection, RF, LR, NB, KNN, SVM, digital image analysis, and CNN for detection of breast cancer after analyzing WDBC, and WSI datasets.

#### 4.3.3 Feature Selection-based Classification

Feature selection is the process that includes the identification of the very relevant and preferred features from multi-dimensional datasets available in the digital public repositories. This process ameliorates the overall performance of different ML classification models. The approach is specifically valuable for patient-related confidential data involving histopathology-based features, statistics, and genes-related data.

#### 4.3.4 Major Techniques and Applications

**i. Recursive Feature Elimination**: This technique recurrently minimizes the least significant features enhancing the performance of the ML model, assuring that only the important attributes should be selected for the classification purposes.

**ii. Principal Component Analysis**: PCA is a feature selection technique for the data transformation to the group of stochastic elements, securing the crucial divergence. This technique has been effectively employed for the dimensionality reduction and duplication of the datasets of breast cancer.

**iii. Evolutionary Feature selection**: EFS is a technique utilized in the area of ML to ameliorate the performance of ML classification models. It applies the evolutionary algorithms (e.g. hlGA, PSO, ICA) for the identification of the subset of attributes that assist in the effective contribution to the anticipation of the accuracy of the classification model. By modeling the process of Darwinism (survival of the fittest), EFS recurrently chooses and combines the relevant attributes to search for the optimal combination through which the performance of the classification is enhanced while reducing the computational complexity.

Saeed et al. 82 has utilized ensemble classification based on MLP neural network, evolutionary algorithm (GA, PSO, and ICA) on WBCD original dataset seeking the classification accuracy of about 98.74%. Roger and the co-authors 18 applied GA and SVM for breast cancer detection over the datasets containing thermal images available in the database for mastology research with infrared image (DMR-IR) and private thermal image database of the Federal University of Pernambuco (UFPE) while achieving the accuracy of 97.18%. Sahar A. 74 employed GA, RFE, rough set feature selection, and PCA for feature selection along with DT, KNN, ANN, SVM, RF, and relief methods for the classification of breast cancer tumors. Khatereh 86 used GA for feature selection in the BreakHis dataset and CNN for classification purposes.

Through exploiting segmentation-based and feature selection-based methodologies, ML classification techniques for the diagnosis of breast cancer have become vigorous and trustworthy. These techniques work together side by side, providing extensive solutions for the analysis of both image-based and feature-based datasets.

### 4.4 Explainable AI and its Necessity for Breast Cancer Diagnosis

As the ML and DL approaches have become complicated to a greater extent, one of the crucial challenges in their real-world acceptance is the meagerness of interpretation and explainability. XAI signifies a collection of approaches developed to cause AI-led resolution more lucid, interpretable, and reasonable for clinicians and patients.

#### 4.4.1 Why is Explainable AI Required?

- **Certitude and Trustworthiness:** Healthcare professionals are more liable to embrace and utilize AI-driven diagnostic approaches if they can comprehend and verify the logic behind the predictions. Black-box DL models such as CNN, mostly lack lucidity, causing physicians reluctant to depend on them for crucial decisions.
- **Administrative Compliance:** Various healthcare administrative departments necessitate that AI-driven methodologies utilized in clinical diagnostics should be explainable and accountable. XAI can assist in ensuring compliance with such rules.
- **Error Detection and Prejudice Alleviation:** Comprehending how AI methodologies derive predictions enables the researchers to recognize potential prejudices and subjectivity, rectify errors, and enhance model impartiality across distinct populations of patients.
- **Enhanced Patient Correspondence:** Providing vivid justifications for AI-based diagnostics allows clinicians to efficiently communicate with convalescents, strengthening logical decision-making and adopting AI-based clinical solutions.

#### 4.4.2 Techniques of XAI

**i. Analysis of Important Features:** Approaches like local interpretable model- agnostic explanations (LIME) and Shapley additive explanations (SHAP) can assist in emphasizing the relevant features (e.g., tumor size, shape, or density) affected a model's prediction.

**ii. Attention Procedure in DL Approaches:** Models such as attention-driven neural networks offer visual interpretation, that makes it simpler to elucidate how a DL model handles clinical images.

**iii. Rule-Driven Models and DTs:** Although DL techniques provide high accuracies, more straightforward rule-based approaches or hlDTs can be utilized in conjunction with them to enhance interpretation.

**iv. Saliency Maps and HeatMaps:** CNNs can produce heatmaps for the visualization of the regions within histopathological images or mammograms that play an essential part in the classification decision.

#### 4.4.3 Real-World Applications of SHAP and LIME

In real clinical settings, SHAP and LIME are mostly utilized to pinpoint the highly influential features or image regions behind the prediction of a model. For example, in mammography or biopsy image analysis, SHAP can visually highlight which parts of a tumor contributed highest to a malignancy prediction assisting radiologists validate or question the AI's outcome.

### 4.5 Performance Metrics for the Comparison

**Figure 10:**
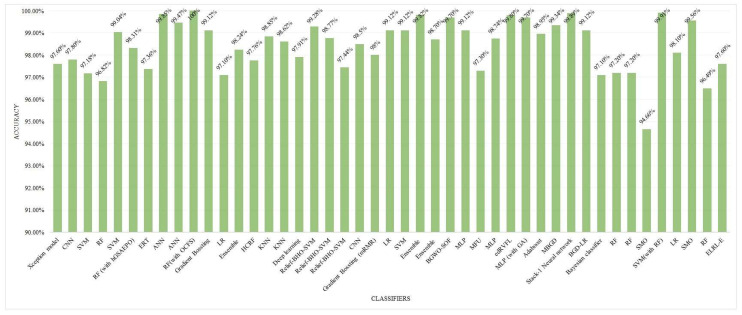
Accuracy for each classification model.

The ML models employed for the diagnosis of breast cancer are frequently evaluated through a number of performance metrics. These performance metrics cater to understanding the algorithms' abilities for the accurate classification of cancerous or non-cancer tumors. The most popular of them is the accuracy. Following is an examination on the basis of Figure 10.

#### i. Accuracy

- **Definition:** This metric depicts the apportionment of properly classified samples (case of true positives and true negatives) into the total number of samples 25, 26 and 32.

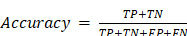

(1)

where TP means true positive, TN is true negative, FP is false positive and FN is false negative. The accuracy of various algorithms ranges between 85 and 100%.

- **Top Performing Algorithms:** The accuracy of GB was 100% proving it an efficient algorithm for the classification of breast cancer tumors into benign and malignant categories. Models such as RF, Xception, and SVM with RF integration attained accuracies of approximately 97% depicted in Figure 10, which reflects their smoothness and vigorousness for classification purposes. The accuracies of every method on different datasets are demonstrated via Figure 10.

#### ii. Sensitivity (Recall or Rate of True Positive)

- **Definition:** This metric provides the ability to measure the model's capability in terms of identification of cancer cases accurately 28, 29, and 34.

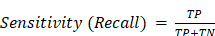

(2)

- **Significance in Breast Cancer Diagnosis:** When the sensitivity of an algorithm is high, it makes sure that the malignant or cancer cases are not bypassed and this is very critical for the early diagnosis of breast cancer and proper treatment. Approaches such as CNN, ensemble techniques, and GB algorithm mostly function in a good manner in terms of sensitivity rate because of their capability to grasp complicated patterns, especially in image-based datasets.

#### iii. Specificity (Rate of True Negative)

- **Definition:** This performance metric measures the model's capability to accurately diagnose the negative cases or benign cases (non-cancerous cases) 39, 40, and 41.

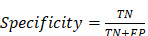

(3)

- **Significance in Breast Cancer Diagnosis:** When the specificity of a model is high, it minimizes the chances of false positive or inaccurate identification ultimately reducing the superfluous biopsies and the strain over patients. Ensemble approaches such as RF and GB are conventionally powerful in attaining an equilibrium between sensitivity and specificity.

#### iv. Precision

- **Definition:** This metric evaluates the number of anticipated positive cases that are in fact in this way 30, 31, and 32.

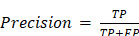

(4)

- **Importance in Breast Cancer Diagnosis:** Escalated precision minimizes the number of false positive rates, which ensures that merely real malignant cases are marked for advanced detection purposes.

#### v. F1 Score

- **Definition:** The harmonic mean of recall or sensitivity and precision is called the F1 score which stabilizes the trade-off between precision and recall 30, 31, and 32.

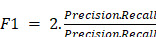

(5)

- **Relativity to Breast Cancer Diagnosis:** When this score is high, it points out a poised performance in the identification of true positive cases and reduction of false positive rates. Approaches with vigorous generalization abilities like GB and Xception algorithm mostly have a good F1 Score.

#### 4.5.1 Performance Metrics Evaluation for Particular Techniques

**Figure 11:**
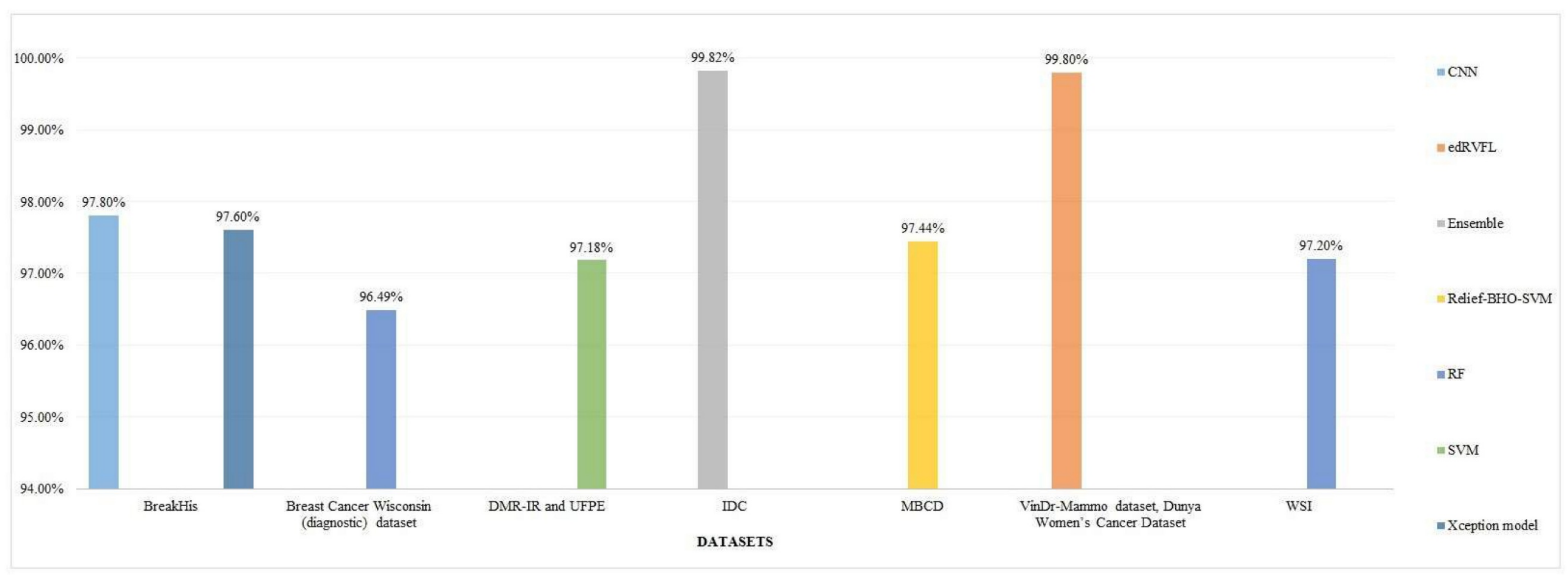
Accuracy for different models for different datasets.

**Figure 12:**
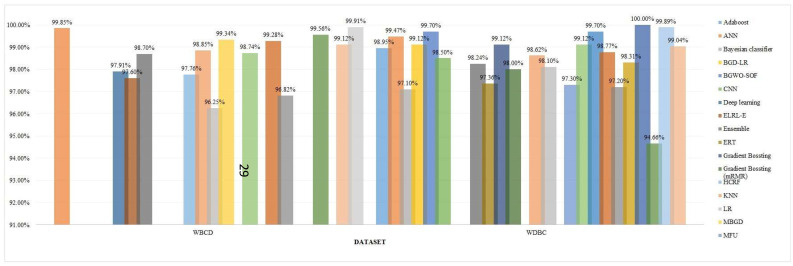
Accuracy for different models using WBCD and WDBC datasets.

- **GB (100% accuracy):** It probably surpasses other models in terms of sensitivity and specificity having an excellent F1 Score. The iterative methodology enables it to perform effectively for various datasets specifically structured datasets such as WDBC or WBCD as shown in Figures 11 and 12.
- **Xception algorithm and RF (97% accuracy):** Both of these models display top performance, where RF performs well in the management of features while the Xception algorithm excels across image-based datasets. RF performs well across IDC and BreakHis datasets while Xception achieves high accuracy in IDC and VinDr-Mammo.
- **SVM (93.95% accuracy):** This algorithm showcases an exceptional performance in terms of binary classification tasks of particularly large-sized datasets such as WDBC. Other performance metrics such as sensitivity and specificity are usually shown as on peak but the number can be varied based on the adjustment of the model parameters.
- **CNN and Other DL models:** These models attain high recall because of their capability of learning complicated patterns, particularly of image-based datasets. Precision showcases minor trade-offs if the sensitivity level of the algorithm is high.
- **LR 93% accuracy):** LR exhibits a reliable performance across less-dimensional datasets having good sensitivity and specificity. It is easy to interpret in relation to DL approaches or ensemble techniques.

Note: For Figure 10 and 11, accuracy values are mainly derived from 10-fold cross validation or test datasets, as reported by respective authors. For Figure 12, performance metrics show test dataset accuracies or average cross-validation scores based source publications.

### 4.6 Discussions

#### 4.6.1 RQ1: How is the performance of various ML models impacted or inflicted by the dataset choice such as WDBC, BreakHis etc. in the diagnosis of breast cancer?

In breast cancer detection research, the credibility of various ML algorithms is substantially affected by the choice of the dataset. Datasets such as BreakHis, WDBC, and WPBC vary enormously in terms of size, complications, and structure, which may have a strong influence on the performance benchmarks of certain models.

There are some large-sized datasets such as BreakHis utilized in 48 and 28 comprising a huge repository of images (histopathological images) which is a source of plenty of data for training DL models like hlCNNs achieving accuracies of more than 99 per- cent.

Contrarily, smaller datasets such as WDBC require vigilant feature selection and are more suitable for conventional ML algorithms like Random Forest (RF) and Support Vector Machines (SVM). These datasets benefit from hybrid approaches that involve preprocessing steps like feature selection prior to classification, often reaching accuracies between 96 percent and 99 percent.

When small sized datasets such as WPBC are employed with complex models, challenges such as overfitting arise. In such cases, hybrid or ensemble methods where combination of feature selection with classification is employed are critical to enhance generalization.

Conclusively, the performance of ML models is essentially related to dataset characteristics. Larger datasets are suitable for DL models, while small tabular datasets favor hybrid approaches. Future research should emphasize on leveraging diversity of the dataset and structure-aware techniques to attain optimal performance.

#### 4.6.2 RQ2: Do the research results contain the prejudice just because of the excessive utilization of prevailing datasets and how this can be alleviated?

There is a considerable bias in breast cancer detection research because of the prevalent use of particular datasets like BreakHis and WDBC. This over-dependency halts the generalizability of models, as they often fail when employed to different or unseen forms of data.

Datasets such as BreakHis continue to dominate the research arena because of their huge size and image-based characteristics. However, models trained specifically on such datasets face performance issues on other datasets, such as clinical or thermal imaging data. For example, CNN-based models that best perform on BreakHis struggle with WDBC due to the differences in format and structure of the data 28.

To mitigate this prejudice, researchers should incorporate multiple variety of datasets and validate ML models across them. Integrating BreakHis with WPBC or thermal imaging datasets ameliorates the generalizability. Generative models like GANs can also be utilized to create synthetic illustrations, assisting in addressing the class imbalance and scarcity of rare cases.

One study showed excellent outcomes when training on BreakHis, but the same model failed to replicate the same performance over WDBC highlighting the significance of cross-dataset validation. Merging datasets like WDBC and WPBC, as done in 74, proves helpful.

In a nutshell, excessive dependency on a few datasets can crook the outcomes and reduce medical relevance. Introducing dataset diversity, carrying out cross-validation across various datasets, and synthesizing the benchmark standards can significantly enhance the fairness and robustness of ML models in breast cancer detection.

#### 4.6.3 RQ 3: What are the determinants that impact the selection of algorithms for the fact finder in regards to breast cancer detection?

Many factors influence the selection of algorithm for breast cancer detection. These include type of the dataset (either image or tabular), dataset size, features structure, model complexity, and resource availability.

Convolutional Neural Networks (CNNs) are mostly selected for image-based datasets like BreakHis because of their strong feature extraction capabilities. Contrarily, algorithms like SVM and RF are prioritized for structured datasets like WDBC, where tabular features are usually relevant.

In researches preferring sensitivity, RF has shown strong performance over tabular datasets. Similarly, CNNs have shown high accuracy on image datasets. However, resource-intensive models like GANs may not be a good choice in the environment with limited computing power. In such cases, simpler models like Decision Trees (DT) and Logistic Regression (LR) provide more practical alternatives.

In clinical settings where interpretability is crucial, models such as SVM and DT are often preferred over complex DL models. For example, 31 used CNNs to achieve 99.78 percent accuracy on image-based data, Conversely, 29 employed SVM on WDBC, reaching 96.82 percent accuracy with engineered features.

Ensemble methodologies such as combining segmentation or feature extraction with DL models further ameliorate performance. Therefore, model selection must take not only accuracy into account but also the elements of deployment and resource constraints.

Conclusively, factors such as dataset type, desired performance metrics, interpretability, and computational cost altogether guide the algorithm selection. Using hybrid strategies, AutoML frameworks, and dataset-aware techniques can enhance performance of diagnosis while keeping robustness intact.

#### 4.6.4 RQ 4: Is there any trade-off between interpretation and accuracy while selecting the algorithm for diagnosis of breast cancer

In breast cancer detection, attaining high accuracy is crucial, however, interpretability is equally important, especially in clinical environment. This often results in a trade- off where interpretable models miss accuracy, and highly accurate models are not transparent.

DL models such as CNNs trained on BreakHis can achieve accuracies more than 99 percent, but they are considered as “black boxes.” This opaqueness restricts their acceptance in clinical settings. Contrarily, simpler models trained on tabular datasets may offer less accuracy but are easier for clinicians to comprehend.

This trade-off offers challenges in model deployment. Healthcare professionals may hesitate to trust such non-transparent models, even if they are accurate. Conversely, relying solely on interpretable models could result into false positives or negatives if accuracy is compromised.

To tackle this issue, hybrid and ensemble approaches have been explored. These models combine several algorithms to balance accuracy and interpretability. For example, in 33, Random Forests offers 100 percent accuracy and are relatively interpretable. Other studies 28, 29, 32 reported top accuracies but do not provide model explanation techniques.

Explainable AI (XAI) tools like SHAP and LIME can assist in making DL models more interpretable. However, their incorporation into research and clinical environment remains scarce. More work is required to make these tools a benchmark practice.

In a nutshell, addressing the accuracy interpretability trade-off is critical. Hybrid methodologies, ensemble models, and XAI integration provide feasible paths forward to ensure reliable and trustworthy diagnostic systems for breast cancer.

### 4.7 Limitations of this Study

While the referenced literature on breast cancer detection techniques provides valuable information, it is important to acknowledge certain limitations and challenges identified in all studies.

- **Restricted Scope of the Dataset Usage:** This review essentially targets the frequently utilized datasets across the literature, clearly ignoring the rising datasets that can provide invaluable insights into breast cancer research.
- **Prejudice in the Chosen Methodologies:** The concentration on specific techniques such as segmentation and feature selection-based techniques might lead to overlooking other nascent methodologies like reinforcement learning.
- **Explanation of ML Models:** Various ML approaches provided varying results for breast cancer detection. The study only looked into their overall performance such as accuracy, and F1 score, without looking at the rationale for their good or bad performance. Looking into their working mechanism might provide better insights.

## 5. Conclusion and Future Direction

The deduction of this study highlights that the performance and generalizability of various ML models are impacted by the dataset choice such as WDBC, BreakHis, etc. in the diagnosis of breast cancer. Large-sized image-based datasets like BreakHis facilitate DL algorithms and the accuracy of more than 99% was attained. Whereas, small-sized tabular-based datasets such as WDBC require cautious feature development and hybrid approaches for reaching sufficient accuracy. While the over-dependency on various datasets incorporates prejudice, restraining clinical applications. The priorities of future research should include the versatility of datasets, incorporating multi-faceted data such as image-based, and genome-based medical data to improve the robustness of the model. For progressing and developing in the area of ML, target of the future studies must be:

- **Explainable AI Development:** The algorithms that provide translucence and interpretability should be preferred which will enable healthcare professionals to comprehend and believe in ML-driven predictions and solutions.
- **Enhancement of Dataset Heterogeneity:** Incorporate more heterogeneous and diversified datasets including changing demographics, medical imaging process, and the properties of tumor.
- **Multi-Dimensional Data Integration:** Image-based data should be combined with the genome, protein-based, and medical data for an inclusive approach to breast cancer detection.
- **Optimization of Efficiency of Resources:** Engineering the compact ML models more suited for deploying in the environment where resources are scarce.
- **Nurturing the Combined Research:** Promoting a diverse setting where the multidisciplinary cooperation among data scientists, radiologists, and oncologists narrows the gap between healthcare and technology.

Alongside the above dimensions, there is another research question arose i.e. what will be the future trend of this research? i.e. How can the simpler algorithms such as hlLR in contrast to the more complex models like DL concerning computational cost and accuracy?

In addition to this, the challenge of the trade-off between the accuracy and the interpretation will remain intact with the elementary models which provide lucidity but do not offer higher accuracies as offered by the DL models. Taking up the XAI, cross- dataset validation, and streamlining approaches are an important breakthrough. A roadmap is provided for progressing the diagnosis of breast cancer in this survey via rational, exact, and understandable ML approaches.

## Figures and Tables

**Figure 5:**
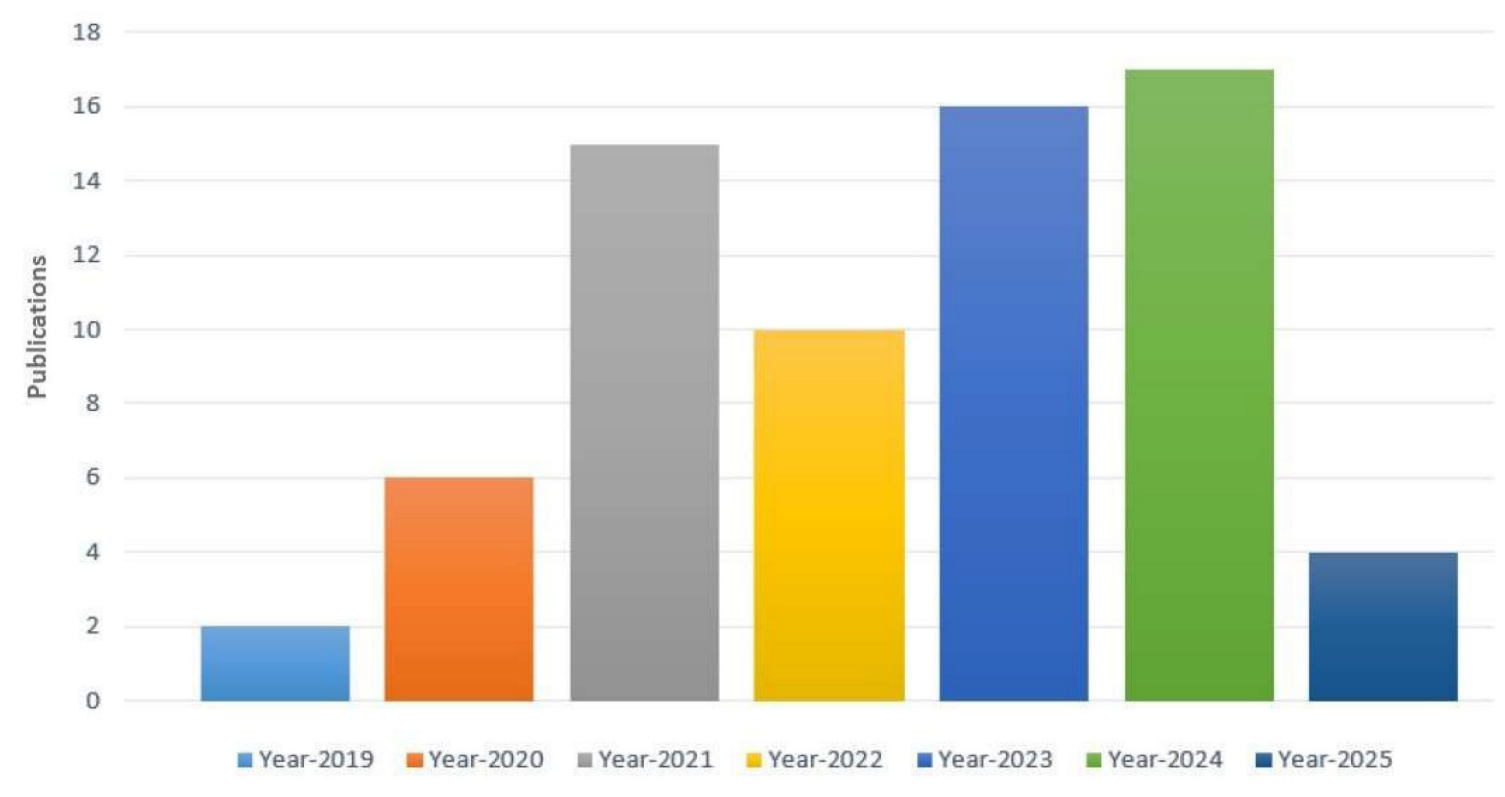
Year-wise publications.

**Table 1:** Comparative analysis of breast cancer detection surveys with respect to research questions addressed in the study.

Ref	Datasets	H-Index	Segmentation	Feature selection	XAI
19	WBCD and only image-based datasets (ultra-sound, histopathology, MRI, etc.)	No	Yes	Yes	No
20	WBCD datasets and image-based	No	No	Yes	No
21	WBCD datasets and image-based	No	No	No	No
22	Image-based datasets only	No	Yes	Yes	No
23	WBCD, WDBC and image-based datasets	No	No	Yes	No
24	Only image-based datasets	No	Yes	Yes	No

**Table 2:** Publisher-wise distribution of papers with corresponding references.

Publisher	Count	Reference Papers
IEEE Access	14	36, 45, 48, 53-63
MDPI	11	18, 34, 49, 64-70
Springer	5	30, 71-75
BMC Series	3	46, 76, 77
Elsevier	3	17, 32
Wiley	3	51, 78, 79
Advances in Artificial Intelligence and Machine Learning	2	66, 80
Archives of Breast Cancer	1	66
International Journal of Advanced Computer Science and Applications	1	28
Asian Pacific Journal of Cancer Prevention	1	29
International Journal of Electrical and Computer Engineering	1	41
Bulletin of Electrical Engineering and Informatics	1	42
International Journal of Integrated Engineering	1	81
Concurrent Engineering Research and Applications	1	52
International Journal of Reconfigurable and Embedded Systems	1	31
International Journal of Image, Graphics and Signal Processing	1	34
Journal of Experimental and Theoretical Artificial Intelligence	1	82
IOP Conference Series Materials Science and Engineering	1	83
Journal of the Nigerian Society of Physical Sciences	1	33
Journal of the Chinese Institute of Engineers	1	37
Jundishapur Journal of Microbiology	1	40
African Journal of Biomedical Research	1	44
Journal of Electrical Systems	1	84
Automation Controls & Engineering	1	85
Simulation	1	86
Neural Network World	1	39
MATEC Web of Conferences	1	43
Jundishapur Journal of Natural Pharmaceutical Products	1	50
